# Lecture on Clinical Medicine, Delivered at the Philadelphia Medical Institute

**Published:** 1838-10-10

**Authors:** W. W. Gerhard

**Affiliations:** Physician to the Philadelphia Hospital, &c.


### CLINICAL LECTURE.

LECTURE ON CLINICAL MEDICINE, *delivered at the Philadelphia Medical Institute, by* W. W. GERHARD, M. D., *Physician to the Philadelphia Hospital*, &c.

### INFLAMMATIONS OF THE HEART.

IN my last lecture I related to you a case of acute inflammation of the pericardium, occurring during the course of acute rheumatism; I then mentioned the physical signs, which were sufficient in themselves for the diagnosis of a case otherwise quite latent. I alluded also to two other cases of acute inflammation of the membranes of the heart, in which the relation between the physical signs and morbid phenomena was perfectly established; and thus afforded you unequivocal evidence of what I had stated in the course of the lecture. We must now prosecute our inquiries still further, and ascertain if there exists any definite connection between ordinary symptoms and inflammations of the membranes of the heart.

Now, the case which you have just witnessed, has proved to you that pericarditis may be entirely latent; that is, no symptoms disclose the existence of the disease to one who is not familiar with the physical methods of exploration. Now, it was once stated that these inflammations were rare, but that when they really occurred the signs were most unequivocal. This was a natural inference derived from a few very severe cases, in which there was extreme functional disorder of the heart and intense orthopnœa. These cases are indeed rare, and may be regarded as altogether exceptional. When the ordinary symptoms are present, they are usually quite violent, while in a very considerable proportion of cases they are entirely absent.

The most marked signs were observed in Robb, to whose case I have so often alluded; now, in him, there were moderate pain in the region of the heart, palpitation, and oppression. The pulse was throughout the greater part of the disease quite regular; it was not very irregular in any of the cases, so that you may conclude that the irregularity of the pulse is quite unusual in heart-disease of an acute kind, especially pericarditis; although it is almost universal in chronic valvular disease. In one other case, there were some local symptoms attributable to the heart; these were acute pain, and a little dyspnœa, but no palpitations sufficiently severe to attract the notice of the patient, who chanced to be of remarkably obtuse sensations. We may therefore conclude in the most positive manner, that acute pericarditis is generally latent, and that it is almost always obscure, if we are reduced to form our diagnosis from the ordinary symptoms alone; while it is readily recognised by its physical signs, which are constant, and with a little attention easily discovered.

Now, you may ask yourselves, of what use is this refinement in diagnosis? The patients did not die of the pericarditis, and it is probable that if the disease had not been examined so minutely, they would still have recovered. I answer, as I always do to such interrogatories,—that the use of no one fact in medicine can be properly estimated when standing alone, but that it must be viewed in connection with the whole train of morbid action and with a series of other facts which bear more or less nearly upon it. When you apply this kind of reasoning to the matter in question, you will be struck with the truth of this remark. Pericarditis and endocarditis are in themselves neither frequently fatal nor very painful; but as you will presently see, they are the causes of numerous diseases of the heart of a chronic character and of a very serious nature, which generally destroy life sooner or later. Hence we should ascertain the existence of pericarditis whenever practicable, and not only inquire into the symptoms, but fix with precision its time of termination; for, if an obscure and semi-acute variety of inflammation should still continue, the probability of the occurrence of chronic disease of the heart is vastly increased, and the patient almost necessarily suffers from it. It therefore becomes your duty not only to check the progress of the disease, but to remove all traces of it, and you must not lose sight of your patient until he is absolutely well. Now, there are no means of watching the course of these diseases, except those derived from physical exploration; and you will at this time find that few are disposed to underrate the value of the practical application of these signs to the study of heart-disease, provided they are really not familiar with their employment. For there is clearly no method of becoming acquainted with the condition of the heart, if we are deprived of the physical signs.

I am the more anxious to insist upon these points, because I believe that we are on the eve of an important train of discoveries in medicine, which can only be brought about by a minute observation of the phenomena of disease, and afterwards by a careful comparison of them, until they become adjusted one to the other, and these scattered facts are, as it were, linked together into one connected chain. These collections of facts then lose their complicated character, and, like all truths which are reached by a laborious process of observation, become as remarkable for their simplicity, as at first sight they were confused and irregular.

The facts relative to the diagnosis of heart-disease, clearly illustrate what I have just stated, and you have at last been able to trace the series of pathological phenomena constituting acute inflammation of the membranes of the heart from their earliest stage, to their termination in organic disease of the valves. You have done this by first acquiring a knowledge of the natural sounds of the heart, and afterwards acquainting yourselves with the morbid sounds heard in cases of extensive organic disease, complicated with alterations of the valves. These latter cases are those in which the morbid sounds are loudest, and are therefore the most suitable for study; and you should commence with cardiac diseases by attending to their well marked cases, before you proceed to the acute inflammations, which are necessarily more obscure. After you have acquired an accurate knowledge of the diagnostic signs, you may then fill up the series of pathological changes, by tracing backwards the history of the causes which you see only at an advanced period, and by watching the termination of the more acute forms of disease which you may have seen at their earlier stages.

You must have drawn your own inference as to the prognosis of these inflammations. You have not seen a single case terminate fatally of eight or ten that you have witnessed during the last six months. Two patients affected with pneumonia, which had passed to the stage of suppuration before their entrance, died. In one of these cases, the pneumonia was evidently the cause of death; in the other, death ensued partly from the suppuration of the lung, and partly from delirium tremens; the pericarditis seemed to have had very little influence in hastening the fatal termination.

Now, your observations during the last six months, have confirmed what the later writers state relatively to the mortality in pericarditis,—but you must look at the prognosis of these diseases in another light; that is, though not immediately fatal, they are apt to terminate in incurable affections of the heart. The probability of this termination varies with the nature of the disease; if the internal membrane of the heart be much inflamed, the valves are almost necessarily thickened and more or less obstructed in their motion; hence we observe the most severe forms of heart-disease, as a very frequent consequence of endocarditis. But if the pericardium be inflamed, the heart will sometimes remain quite free from organic disease, and the patient will not necessarily suffer from palpitations, or other functional disorders of this organ. I observed a case of pericarditis early in the summer of last year, and traced it through its various stages to entire recovery: more than a year had elapsed, when I was informed of the death of the patient from an acute inflammation of the brain, in nowise connected with the heart. The cavity of the pericardium was completely obliterated by very close cellular adhesions, which had formed during its acute inflammation, but the heart was quite natural in its appearance, except that it was somewhat smaller than usual. In other cases, however, the heart becomes enlarged and dilated, even when very little or no inflammation of its internal membrane had occurred. These cases of chronic disease of the heart following inflammation, usually terminate in dropsical effusions into the serous cavities and cellular membrane.

The causes of cardiac inflammation are now well known. By far the most frequent one is acute articular rheumatism; you did not observe a single severe case of this disease, in which the inflammation of the heart did not occur, and there is no doubt that it is very rarely absent in any case attended with much fever. When the rheumatism is quite moderate, the cardiac complication may exist; but it is by no means as common as in those cases in which most of the larger joints are affected, and in which the febrile excitement is high. For our knowledge of this complication we are certainly indebted to Dr. Bouillaud,—I mean for our positive knowledge of its great frequency, for the fact has been often pointed out in a general way. Rheumatism is by no means the only cause; exposure to cold, and other causes of serous inflammations, frequently produce inflammation of the membranes of the heart, either as idiopathic affections, or as mere complications of pleurisy and pneumonia. In the latter case, it certainly increases the severity of the disease. Chronic disease of the heart favours the developement of acute inflammation of its membranes, much on the same principle that tubercular or other chronic disease of the lungs is a frequent exciting cause of pleurisy. I need not detain you longer upon the causes of these affections, which are in other respects identical with those of all the serous inflammations.

I have not alluded to the inflammation of the proper tissue of the heart; like all the muscular tissues, it is very frequently the seat of rheumatic irritation, particularly when it is previously enlarged or dilated. You will find no difficulty in recognising this form of disease, from its sudden and severe pain, and the palpitation which immediately occurs, but you will not find that the physical signs of inflammation of the membranes are present. Now, from their absence, combined with the occurrence of the functional disorder, you may discover the nature of the disease. Suppurative inflammation of the heart is so rare, that you may safely leave it out of the calculation; extensive as my acquaintance with pathological anatomy has been, both in Europe and in this country, I have never met with a case in which there was pus in the proper tissue of the heart.

I have now to speak of a very interesting part of this subject,—the therapeutics. It will be the more interesting to you, because I take it for granted that you have not neglected the opportunities which you have enjoyed of becoming familiar with the symptoms of the disease, which of course render therapeutics much more certain in their application. I will first speak of the treatment of pericarditis. The pericardium is situated, as you well know, very near the exterior of the chest,—so that we can apply our local remedies much more closely than in cases of deeply seated inflammation; we thus have every facility for using local depletion. I place this remedy first, because it is undoubtedly the most useful. I have studied this part of the subject with great attention, and have no doubt whatever that local bleeding is much more efficient than general. I mean, that however useful venesection may be in preparing the system for local bleeding and diminishing the activity of the circulation, it has a less unequivocal action upon the course of the disease than cupping over the præcordia. Nevertheless, you will find that the same general rules, as to the propriety of blood-letting, hold good in pericarditis as in other inflammatory diseases where there is an active circulation,—you may, I can almost say, you must open a vein, and abstract a sufficient quantity of blood to diminish the violence of the heart's contraction, and relieve the engorgement of the lungs. These conditions interfere greatly with the recovery of the patient, and add much to his present suffering; but after you have relieved the oppression, and have reduced the activity of the pulse, or rather have rendered it softer and more developed, the object of blood-letting is attained in pericarditis, and you have now to rely in great part upon local depletion, particularly by means of cups, which have certainly many advantages over leeches.

You have witnessed the effects of cupping during the past course. I was anxious, as far as possible, to subject this means of treatment to something like a definite rule. Now, this could be done only in one way,—that is, by applying cups in such a manner as to take very various quantities of blood, and then carefully noting the effects of each application. When I applied two cups only, and had but three or four ounces of blood taken away, the patient was not at all or very little benefitted, if he was moderately robust. Indeed, you have sometimes found the uneasiness a little increased; but when seven or eight ounces were taken from three or four cups, the patient was invariably relieved, and in a very short time after their application. Now, from this observation, which was verified in the strictest manner, you may understand how necessary it is to be precise in your application of therapeutics; a minuteness, which, at first sight, may seem insignificant, made all the difference between a good result, and one that was either nugatory or positively mischievous.

This attention to minutiæ in therapeutics is perfectly well understood by surgeons, who never attempt to treat a case even of simple wound, without most carefully adjusting the lips of the wound, bringing it accurately together, and what is most important, allowing free exit to pus if any should form. If the adhesive strips be applied very carefully and well, a single error in closing the orifice too completely, may destroy all prospect of speedy cure. Now in our more difficult medical therapeutics, the same rule holds good, and if we find it impossible to arrive at the certain results which are yielded by simple external injuries, we may nevertheless do much towards removing the uncertainty of our therapeutics, by carefully following out the indications which are based upon well ascertained grounds.

The cupping should be repeated if pain should continue, or if the effusion is known from the physical signs to be upon the increase, but you are not to repeat the cups because a grating sound should still be heard over the heart. For this grating sound is in reality a mere consequence of the partial adhesion of the two surfaces of the pericardium together, and when it appears after the effusion has been almost removed, it is one of the most unequivocal signs of approaching cure. If the creaking continue when all the other general and local signs have ceased, it becomes the more necessary to abstain from further active measures of any kind.

You have seen me treat cases of acute pericarditis complicating rheumatiam, by cups applied along the spine, according to the method so successfully recommended by Dr. J. K. Mitchell of this city. The cups were severally applied in this manner to the patient whose case was mentioned at the last lecture; now while this treatment proves very useful in many cases of rheumatism, it is not without importance as regards the pericarditis, which is at least as much relieved as by general bleeding to the same amount.

I have rarely applied blisters during the past summer, simply because I have not found them necessary, for the patients recovered very well without the necessity of resorting to a disagreeable remedy. But you will find them very useful in pericarditis, if the case should linger and the effusion remain considerable. They act in this case in the same manner as in other serious inflammations, which are always very speedily relieved by blisters, if they are near the surface to which the blister is applied. Now this rule of contiguous inflammations being very speedily relieved by blistering, applies with peculiar force to pericarditis; as the whole pericardium is near the skin, and that portion of it which is most superficial is most liable to inflammation, and is always more inflamed than the surface which is most distant. When I apply blisters in pericarditis, it is chiefly in the chronic or sub-acute cases, and I then prefer using small ones very frequently to applying a single large one. This method is more effectual and less irritating. With the other counter-irritants I have no experience in true pericarditis.

Numerous internal remedies have been recommended in the treatment of pericarditis. Now the greater part of these remedies are designated by those authors who regard pericarditis as a disease which is of extreme immediate danger; hence an exaggerated importance may be attached to some of them. You are, however, bound never to leave a case of pericarditis, particularly if it be severe, without general treatment; for these internal remedies will certainly diminish much of the suffering and inconvenience of the disease if they are insufficient to arrest it suddenly. Here you will find me considerably embarrassed; for, desirous as I am to impress upon you the necessity of using these remedies which are certainly useful, I feel at times some reluctance in laying the same stress upon others whose advantages are more uncertain. Still, by carefully analyzing the effects of these internal remedies, we may do something towards removing this uncertainty.

First, you have seen me use remedies which are generally regarded as serviceable in checking inflammation wherever seated. That is, the nauseants and diaphoretics, particularly tartar emetic. In the same class we may include the Dover's powder, in which opium is so useful in connection with ipecacuanha. Mercurials, which are so useful in the management of chronic serous inflammation with considerable effusion, I have never found necessary in pericarditis. You must not expect from any of these remedies as much obvious advantage as from bloodletting; still I am entirely convinced of their utility from a variety of facts which are with difficulty introduced into a lecture. I, therefore, place the patient under the influence of an opiate and a nauseant, such, for example, as two or three grains of Dover's powder, with a sixth or an eighth of a grain of tartarized antimony, every two or three hours. Opium in larger doses you are aware is much recommended in the treatment of acute articular rheumatism, which is of course very often complicated with pericarditis. Now, from my own observation, I cannot concur with some highly respectable physicians of New England, in regarding opium as capable of arresting acute rheumatism and still less the complication of pericarditis which so frequently attends it. As to the propriety of an occasional increase in the dose of opium towards night, I have no specific directions to give but must leave it to your own tact and discretion.

There is another remedy, which has obtained to some extent the reputation of a specific in diseases of the heart. I allude, of course, to digitalis. Now, although this remedy is often grossly abused, you will find no substitute for it in the acute inflammation of the heart, and in some of the subacute varieties of inflammation which occur from time to time during the course of its chronic diseases. I am more partial to its use in those varieties of pericarditis which are not connected with rheumatism, than in that which forms a mere part of this latter disease. This preference may arise from habit, although I believe that it is founded upon experience. I now usually prescribe the digitalis in connection with Dover's powder; say, half a grain of the former with three grains of Dover's powder, every two or three hours. When there is much effusion the digitalis is most useful, from its diuretic powers, but in all cases of acute inflammation of the heart, its sedative properties are extremely important.

Besides these general rules of treatment, there are a multitude of less important adjuvants; such as warm cataplasms of hops or other similar remedies over the præcordial region. These are all more or less useful, and must often be prescribed together with those hygienic precautions which must occur to most of you, at the close of pericarditis. In its acute stage there is but little immediate danger, and the patient himself is usually disposed enough to attend to the necessary precautions, but when threatened with a chronic disease of the heart, few are willing to conform to the restrictions which are then indispensable.

I have scarcely alluded to the difference in treatment required by endocarditis and pericarditis. It is slight; but still the treatment should not be precisely similar in both these affections. You will find that endocarditis is less certainly relieved by local bleeding than the inflammation of the external membrane of the heart. General bleeding is more necessary and we use digitalis more constantly than in the latter disease. If you bear in mind these few points, you will have no difficulty in managing ordinary cases of endocarditis, but in the more severe forms, the patient is much prostrated, symptoms of asphyxia appear, and the extremities are cold from the slowness of the capillary circulation. The pulse is also feeble and often irregular. In these cases you will find the treatment very difficult, and it will require much tact on your part.

You will be most successful by bearing in mind two leading indications, which are often necessary in therapeutics. That is, you have at the same time to excite the capillary circulation, and restore heat to the surface, while you attempt to tranquillize the circulation, and to relieve the heart of those accumulations of blood which are so apt to take place in endocarditis. Now the first indication is answered by applying dry heat to the surface, giving assafœtida and Hoffman's anodyne, or some other ethereal preparation. I usually direct half a drachm of Hoffman's anodyne or sulphuric ether, with five grains of assafœtida, every half hour, until the paroxysms should diminish; at the same time, I apply flying sinapisms over the extremities, and particularly between the shoulders, with mustard foot-baths. These remedies will always succeed, if the patient be not very near death, in answering your indications, and will enable you to resort to bleeding or to a free cupping over the heart. You will find that dry cups and sinapisms, which act especially upon the nervous system, are most beneficial upon the spine,—while scarified cups are certainly much more effectual when applied over the heart itself.

After the circulation is thus restored to a proper equilibrium, and the dyspnœa is somewhat diminished, you may resort to the treatment already recommended for endocarditis. If the disease be complicated with pneumonia, these paroxysms of extreme suffering and prostration are the more apt to occur; and general bleeding, if directed without observing the precautions which I have just mentioned, is decidedly injurious; it favours the formation of coagula in the heart, which are the ordinary cause of death.

Another cause of death arises from the injury done to the internal membrane of the heart, and particularly to the valves. I have seen the valves completely eroded by ulceration, so that their functions were interrupted and death occurred suddenly, much in the same way as in chronic disease of the heart. In all cases, the disease should be regarded as a treacherous affection, and your watchfulness should not cease until the absolute recovery of the patient.

